# Dynamin 2 is essential for mammalian spermatogenesis

**DOI:** 10.1038/srep35084

**Published:** 2016-10-11

**Authors:** Kate A. Redgrove, Ilana R. Bernstein, Victoria J. Pye, Bettina P. Mihalas, Jessie M. Sutherland, Brett Nixon, Adam McCluskey, Phillip J. Robinson, Janet E. Holt, Eileen A. McLaughlin

**Affiliations:** 1 School of Environmental and Life Sciences, University of Newcastle, Callaghan, NSW 2308, Australia; 2 PRC in Chemical Biology, University of Newcastle, Callaghan, NSW 2308, Australia; 3 School of Biomedical Sciences & Pharmacy, University of Newcastle, Callaghan, NSW 2308, Australia; 4 Cell Signalling Unit, Children’s Medical Research Institute, University of Sydney, Sydney, NSW 2145, Australia; 5 School of Biological Sciences, University of Auckland, Auckland 1010, New Zealand

## Abstract

The dynamin family of proteins play important regulatory roles in membrane remodelling and endocytosis, especially within brain and neuronal tissues. In the context of reproduction, dynamin 1 (DNM1) and dynamin 2 (DNM2) have recently been shown to act as key mediators of sperm acrosome formation and function. However, little is known about the roles that these proteins play in the developing testicular germ cells. In this study, we employed a DNM2 germ cell-specific knockout model to investigate the role of DNM2 in spermatogenesis. We demonstrate that ablation of DNM2 in early spermatogenesis results in germ cell arrest during prophase I of meiosis, subsequent loss of all post-meiotic germ cells and concomitant sterility. These effects become exacerbated with age, and ultimately result in the demise of the spermatogonial stem cells and a Sertoli cell only phenotype. We also demonstrate that DNM2 activity may be temporally regulated by phosphorylation of DNM2 via the kinase CDK1 in spermatogonia, and dephosphorylation by phosphatase PPP3CA during meiotic and post-meiotic spermatogenesis.

The process of mammalian spermatogenesis gives rise to mature male gametes and arguably represents one of the most complex differentiation processes in developmental biology. Germ cell maturation begins shortly after birth with pre-spermatogonial gonocytes differentiating into a self-renewing stem cell population that produce mitotic spermatogonia. Following clonal amplification, these spermatogonia enter meiosis to form primary spermatocytes, which in turn proceed through two rounds of division to produce haploid round spermatids. These spermatids undergo spermiogenesis, an extraordinary phase of cytodifferentiation, culminating in the production of the specialized spermatozoon^1^. Owing to its complexity, this process is tightly regulated through spatial and temporal gene and protein expression. Among the many proteins that have been implicated in this process, preliminary evidence indicates that the dynamin family of large GTPases may support several aspects of spermatogonial amplification, meiotic cell differentiation and spermatozoa development^2^. The dynamins fulfil these roles through regulation of critical membrane fission and fusion events. Such functions are attributed partially to the ability of dynamin to assemble into helical polymers at the neck of a budding vesicle. A GTP hydrolysis-dependent conformational change in dynamin then promotes fission of the underlying tubular membrane in order to generate a free endocytotic vesicle^3^. In addition to this, dynamin has also been shown to act as a microtubule binding protein^4^,^5^, potentially regulating microtubule instability and function^6^,^7^, as well as controlling cytokinesis in dividing cells^8^. The mammalian genome contains three dynamin genes (*Dnm1, Dnm2* and *Dnm3*) encoding the three ‘classical’ members of the family, dynamin 1 (DNM1), dynamin 2 (DNM2) and dynamin 3 (DNM3). All three proteins share the same domain organization and have over 80% identity, but display distinct expression profiles. DNM1 is predominantly expressed in neuronal tissue, and is critically required for the efficient recycling of synaptic vesicles^9^,^10^. Due to the severity of synaptic transmission defects, DNM1 null mice are limited to an average life span of 2 weeks^9^. DNM2 is expressed ubiquitously throughout the body, with knockout of this gene resulting in early embryonic lethality, a phenotype that aligns with its purported housekeeping functions^11^,^12^. In contrast, DNM3 is predominantly restricted to the brain (although at much lower levels than DNM1), testis and lung^13^,^14^. However, DNM3 knockout mice fail to exhibit any obvious neurological or fertility defects^14^.

Within the testis, the dynamins have been shown to differentially localize to both germ cells and Sertoli cells, with DNM1 and DNM2 expressed uniformly in pachytene spermatocytes and becoming restricted to the developing acrosome in round spermatids^2^,^15^. In contrast, DNM3 demonstrates a punctate pattern of localization in these cell types, with decreasing expression in the more advanced stages of germ cell maturation^2^,^16^. In addition, both DNM2 and DNM3 have been associated with the blood-testis barrier and have been implicated in facilitating the endocytic migration of spermatocytes from the basal to the adluminal compartment^17^. However, the precise mechanisms through which dynamins support testicular function and spermatogenesis remain to be fully elucidated. This study employed the use of a conditional knockout model to define the role of DNM2 in supporting spermatogenesis.

## Results

### DNM2 is expressed at all stages of male germ cell development

**Figure 1:**
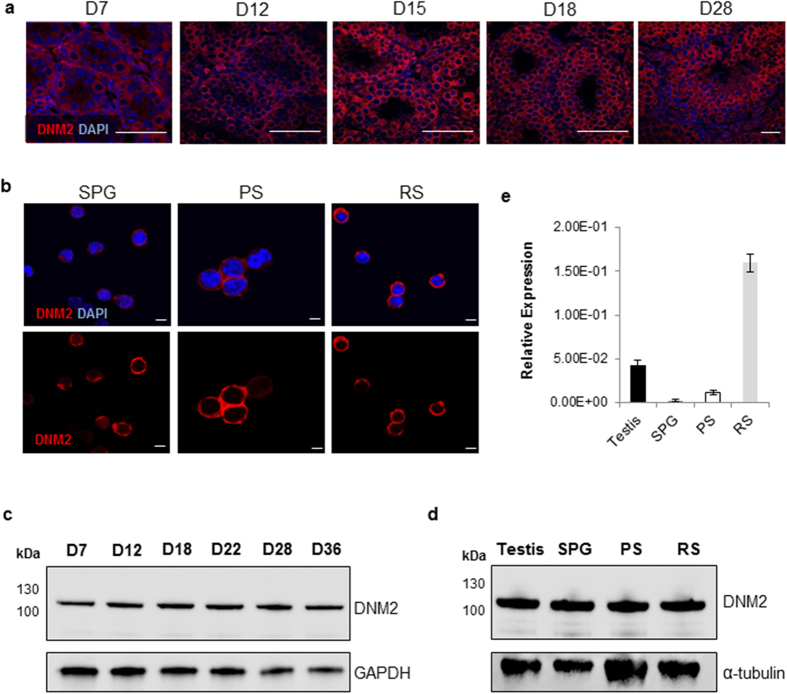
DNM2 expression in mouse testes and male germ cells. (**a**) Fixed tissue sections from neonatal (D7-D18) and young adult (D28) mice testes were probed for DNM2 protein expression using immunofluorescence. The sections were counterstained with the nuclear marker DAPI and visualized using epifluorescence microscopy. (Scale bar = 50 μm). (**b**) Isolated SPG, PS and RS were fixed and DNM2 protein expression was examined using immunocytochemistry (Scale bar = 5 μm). (**c**) Representative immunoblots of DNM2 expression in whole testis from D7-D28. GAPDH was used as a loading control. (**d**) Representative immunoblots of DNM2 expression in isolated SPG, PS and RS compared to whole testis, with α-tubulin was used as a loading control. (**e**) qPCR analysis of *Dnm2* in whole testis, SPG, PS and RS in C57Bl/6 males. Data are presented as mean relative expression to *Cyclophilin A.* (***p < 0.001, **p < 0.01 Student’s *t*-test ± SEM).

Expression of DNM2 during spermatogenesis, was characterised by immunofluorescence in testis sections sampled from post-natal mice aged between 7 (D7) to 28 (D28) days, with each time point selected to characterise key developmental phases during the first wave of spermatogenesis. DNM2 protein was successfully localized in germ cells at all stages of maturation; initially appearing around the periphery of the cytoplasm of spermatogonia (SPG) (D7), pachytene spermatocytes (PS) (D12-18) and round spermatids (RS) (D28) (Fig. 1a). This localization pattern was further confirmed by immunocytochemical analysis performed on isolated populations of SPG, PS and RS, whereby DNM2 expression was detected uniformly throughout the cytoplasm of these cells (Fig. 1b). Immunoblotting confirmed that the overall levels of DNM2 protein expression do not change significantly between D7 and D36 in the testis (Fig. 1c), nor do they differ between isolated populations of SPG, PS or RS (Fig. 1d). In contrast, *Dnm2* gene expression was shown to be significantly higher in isolated RS compared to expression in SPG, PS or whole testis (Fig. 1e).

### Ablation of DNM2 results in arrested spermatogenesis and infertility

**Figure 2:**
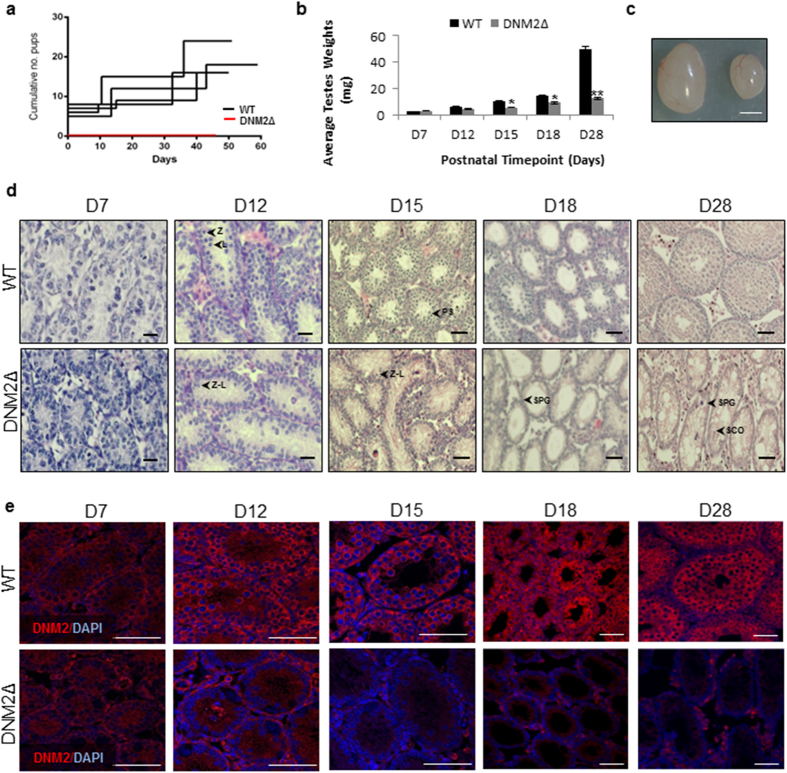
Infertility in DNM2Δ males is a consequence of germ cell depletion before adulthood. (**a**) Graph represents the cumulative number of pups born to mated DNM2Δ or wildtype (WT) males, where Day 0 equals the day of the first litter. (**b**) Mean testis weight for WT and DNM2Δ mice between D7 and D28. (**c**) WT and DNM2Δ testes (D28) imaged immediately following recovery (Scale bar = 2 mM). (**d**) Histological cross sections stained with H&E of WT and DNM2Δ testes between D7 and D28. (Scale bar = 20 μm; L, leptotene; Z, zygotene; Z-L, zygotene/leptotene; PS, pachytene spermatocyte; SPG, spermatogonia; SCO, Sertoli cell only). (**e**) Immunofluorescence probing for DNM2 in WT and DNM2Δ testes between D7 and D28. Sections were counterstained with the nuclear marker DAPI and visualized using confocal microscopy (Scale bar = 50 μm) (*p < 0.05, **p < 0.01, ***p < 0.001 Student *t*-test ± SEM).

Having established that DNM2 is expressed ubiquitously throughout germ-cell development, we next examined the consequences of germ-cell specific knockout of *Dnm2*. To achieve this, we generated a conditional *Dnm2* knockout under the control of the *Ddx4* promoter to eliminate DNM2 (DNM2Δ) protein during mitotic arrest at embryonic day 15^18^. The DNM2Δ males produced (Fig. S1) were mated with wildtype (WT) C57BL/6 females for approximately 3 months to determine the impact of this mutation on male fertility. In contrast to their wild type (WT) littermates, which produced at least 3 litters over this period, the DNM2Δ males were completely infertile, producing no litters (Fig. 2a). Examination of neonatal and juvenile male testes revealed no differences in fresh tissue weights at D7 and D12, however testes from DNM2Δ males at D18 or older were significantly smaller (Fig. 2b,c). Histological examination revealed that D7 DNM2Δ testes exhibited a similar phenotype to controls (Fig. 2d). At D12 meiotic leptotene and zygotene stage cells were clearly present in controls, identified by punctate intense chromatin staining in the nucleus, but few of the cells were observed in DNM2Δ testes. By D15 when mid-pachytene spermatocytes were evident in controls, DNM2Δ testes possessed few cells of pachytene-like morphology, with the tubules depleted of the majority of germ cells (Fig. 2d). The phenotype of germ cell loss and developmental arrest was overt at D18, with the testes of DNM2Δ animals possessing only a single row of SPG-like germ cells remaining in the basement compartment of the seminiferous tubules. At D28, the effect on germ cell development was catastrophic, with the tubules containing very few to no SPG, with the morphology of the remaining Sertoli cells also severely affected (Fig. 2d). In association with this, DNM2 expression was lost from germ-cells by D7 and thereafter restricted to Sertoli cells (Fig. 2e). Importantly, the expression of DNM1 (assessed via immunofluorescent labelling of phosphorylated forms DNM1p774 and DNM1p778) and DNM3 protein were unchanged in the DNM2Δ testis (Fig. S2). Taken together, these data indicate that the absence of DNM2 leads to a progressive age-dependent loss of SPG preceding meiotic arrest during the first wave of spermatogenesis. Further, neither DNM1 nor DNM3 are able to functionally compensate for the loss of DNM2 in these cells.

### Ablation of DNM2 results in delayed and reduced spermatogonia proliferation

**Figure 3:**
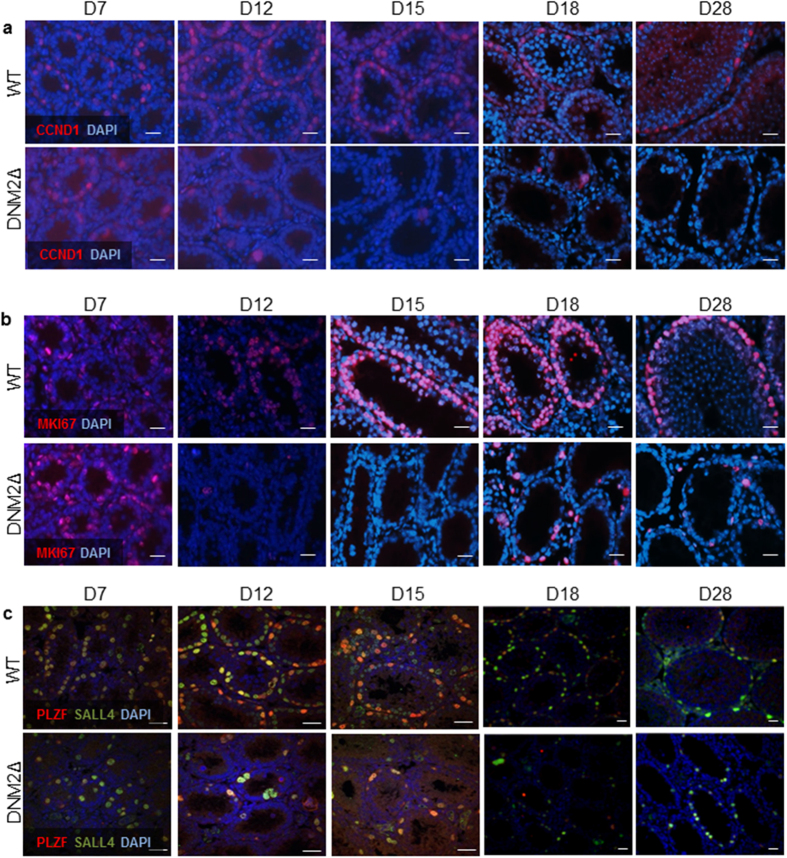
Reduced expression of spermatogonia proliferation markers in DNM2Δ testes. Fixed tissue sections from WT and DNM2Δ testes between D7 and D28 were examined using immunofluorescence. The sections were counterstained with the nuclear marker DAPI and visualized using epifluorescence microscopy or confocal microscopy. Expression of (**a**) spermatogonia proliferation marker cyclin D1 (CCND1) (Scale bar = 20 μm), (**b**) proliferation marker MKI67 (Scale bar = 20 μm) and (**c**) spermatogonial stem cell markers PLZF and SALL4 (Scale bar = 20 μm).

As SPG undergo several rounds of mitotic division, we hypothesized that DNM2Δ SPG may have a proliferation defect, accounting for germ cells loss in the adult. Thus, immunofluorescence was performed using a marker of proliferating SPG, cyclin D1 (CCND1), which is present at highest levels at the G1 and G2 phases of the cell cycle in differentiating SPG^19^,^20^,^21^. CCND1 expression was observed in SPG of WT and DNM2Δ testes at D7, but was significantly reduced by D12 before being virtually undetectable by D15 (Fig. 3a) in DNM2Δ testes. CCND1 loss suggested mitotic cell quiescence may be occurring in the absence of DNM2. To further confirm this hypothesis, we examined an additional marker MKI67, which is expressed during all stages of the cell cycle in dividing cells^22^. Similar to CCND1, MKI67 was considerably depleted in the neonatal testes of DNM2Δ animals from D15 onwards (Fig. 3b), indicating mitotic cell cycle arrest.

To determine if such defects could be attributed to perturbation of the self-renewal and maintenance properties of the SPG stem cell population, we also examined the expression of promyelocytic leukaemia zinc finger (PLZF), a SPG-specific transcription factor and important regulator of SPG stem cells, and SALL4, which is uniquely expressed in undifferentiated SPG. Similar to CCND1 and MKI67, down-regulation of PLZF/SALL4 expression was observed in the tubules of the DNM2Δ testis by D12 onwards (Fig. 3c). Indeed, by D18 only a small subset of SPG were identified to be expressing SALL4 and PLZF. From these data we infer that the establishment and/or maintenance of the SPG stem cell pool is perturbed by the loss of DNM2, resulting in a reduced number of stem cells differentiating into proliferating SPG capable of reaching mitotic exit and entering into meiosis.

### Progression through meiosis is perturbed in male germ cells lacking DNM2

**Figure 4:**
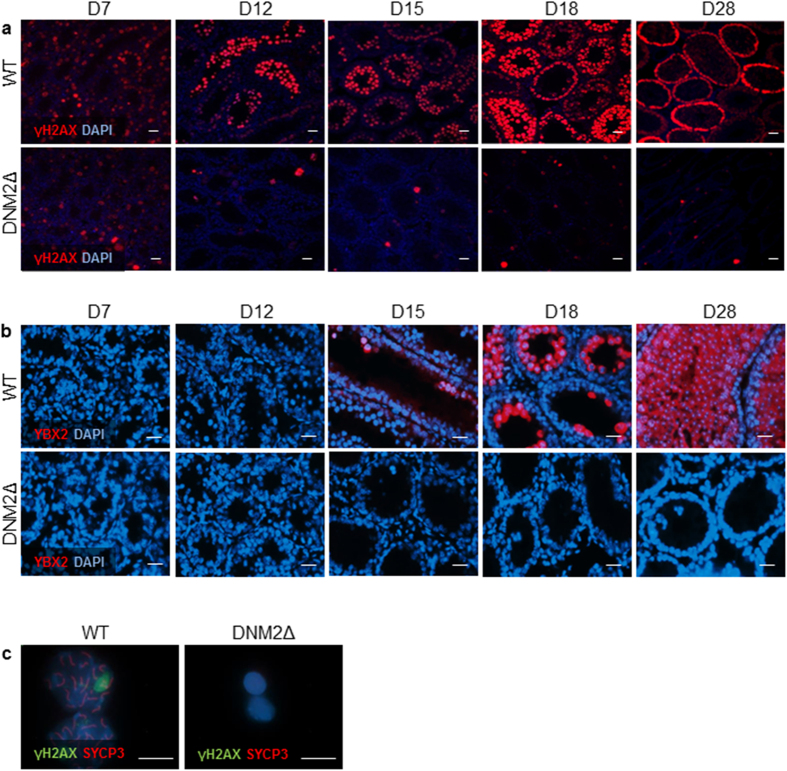
Reduced expression of meiotic markers in DNM2Δ testes. Fixed tissue sections from WT and DNM2Δ testes between D7 and D28 were examined using immunofluorescence. The sections were counterstained with the nuclear marker DAPI and visualized using epifluorescence microscopy or confocal microscopy Expression of (**a**) γ-H2AX, a marker of meiotic DSBs (Scale bar = 20 μm) and (**b**) meiotic marker YBX2 (Scale bar = 20 μm). (**c**) Meiotic cell spreads with immunofluorescent staining of γ-H2AX and SYCP3 in pachytene spermatocytes isolated from WT and DNM2Δ testes (Scale bar = 20 μm).

Notwithstanding the observed abnormal and delayed SPG proliferation, a proportion of SPG in DNM2Δ males are capable of entering meiosis. However, these germ cells fail to progress into PS (Fig. 2). To characterize the germ cell defects in these mice, we examined markers of early meiotic events. The phosphorylation of H2AX to γ-H2AX is one of the earliest cellular responses to the formation of DNA double strand breaks (DSBs)^23^. However, meiotic recombination in mice is initiated by programmed DSBs^24^, and therefore γH2AX should be present in spermatocytes that have correctly entered meiotic prophase I. In WT controls, γH2AX was strongly labelled in preleptotene and zygotene spermatocytes from D7 through to D28. In DNM2Δ testes however, γH2AX staining was significantly down-regulated, suggesting the loss of early meiotic germ cells (Fig. 4a). Examination of YBX2, which acts as an mRNA stabilizer and a transcription factor of protamine genes during nuclear condensation in late stage spermatids^25^,^26^, revealed expression in spermatocytes at D15 in WT testes, increasing at D18 and D28 upon spermatid differentiation. In contrast, no YBX2 labelling was observed in the testes of DNM2Δ animals (Fig. 4b). The absence of such markers indicates that, of the few spermatogonia that enter meiosis during the first wave of spermatogenesis in DNMΔ males, none of these cells are able to complete this process and transition into post-meiotic RS.

To examine more closely the fidelity of early meiosis in these late-entry DNM2Δ spermatocytes, we performed meiotic cell spreads and assessed the progression of recombination by visualizing synaptonemal complex (SC) assembly. The SC scaffold begins to assemble after DSB formation (leptotene stage), and can be visualized by the gradual deposition of the axial/lateral element synaptonemal complex protein 3 (SYCP3) along the length of the chromosome. Meiotic spreads from WT pachytene spermatocytes isolated at D18 displayed SYCP3 staining patterns characteristic of pachytene meiotic stages (Fig. 4c). In contrast, none of the spermatocytes isolated from DNM2Δ testes displayed any SYCP3 or γH2AX staining (Fig. 4c). These observations suggest that DNM2Δ germ cells fail to undergo proper DSB formation and, as a consequence, synapsis is not completed in these cells.

### Loss of DNM2 results in germ cell apoptosis and disruption of DNA repair mechanisms

**Figure 5:**
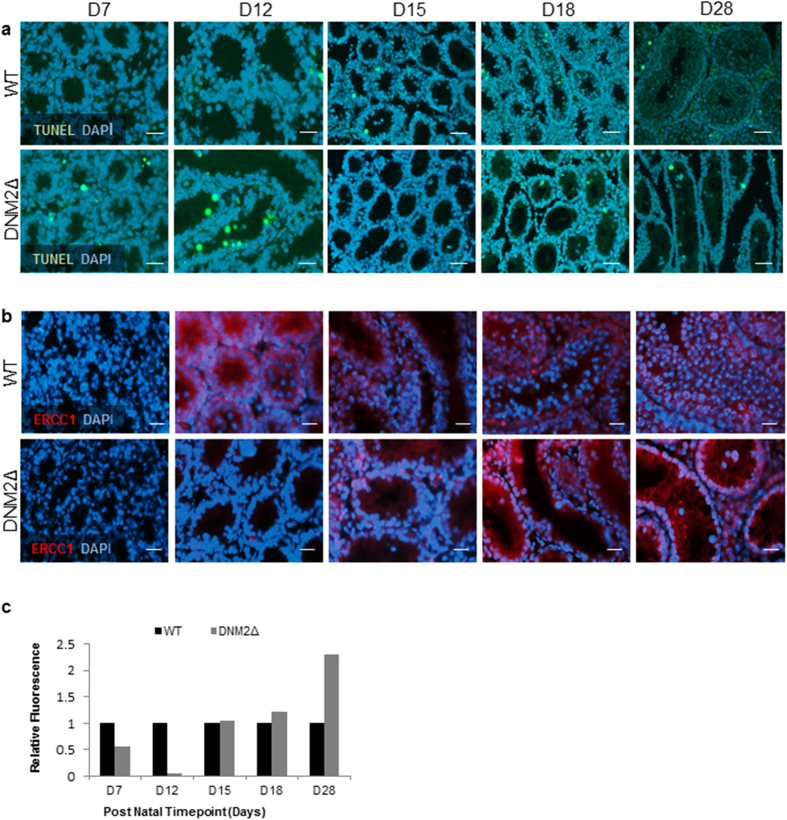
DNM2Δ affects DNA damage and repair pathways. (**a**) Representative images of TUNEL assay performed on WT and DNM2Δ testes between D7 and D28. Green staining indicates cells undergoing apoptosis (Scale bar = 50 μm). (**b**) Immunofluorescent staining of DNA repair protein ERCC1 in WT and DNM2Δ testes between D7 and D28 and counterstained with DAPI. Visualized with epifluorescence microscopy. (**c**) Comparison of the relative DNM2 fluorescence in WT and DNM2Δ testes between D7 and D28 analyzed using Image J software package (*p < 0.05, **p < 0.01, ***p < 0.001 Student *t*-test ± SEM; Scale bar = 20 μm).

Germ cells that have undergone aberrant meiosis or have damaged DNA are eliminated from the testes via apoptosis^27^. To determine if the loss of germ cells observed in DNM2Δ testis was by apoptosis, tissue sections were assessed for TUNEL. In WT mice at all the time points, infrequent TUNEL immunopositive germ cells were observed close to the basement membrane, as is consistent with previous reports indicating that most cells undergoing apoptosis in the normal testis are mitotically active type A SPG^28^. In contrast, although there were considerably fewer germ cells present than in the controls, there were significantly more TUNEL positive germ cells per tubule at D12 in DNM2Δ testis (Fig. 5a). Interestingly, the DNA repair protein ERCC1, which is normally most highly expressed in meiotic cells, was significantly down-regulated at D12 in DNM2Δ testis compared to WT controls (Fig. 5b,c). This indicates not only the failure of the cells to enter and progress through normal meiosis, but also a failure to repair the induced DNA damage. In this context, it is notable that the expression of ERCC1 in DNM2Δ testis is similar to those in WT testis by D15, but was increased by D28 (Fig. 5b,c), potentially indicating attempts to repair DNA damage in the remaining spermatogonia.

### CDK1 is a potential DNM2 phosphorylating kinase in mouse testes

**Figure 6:**
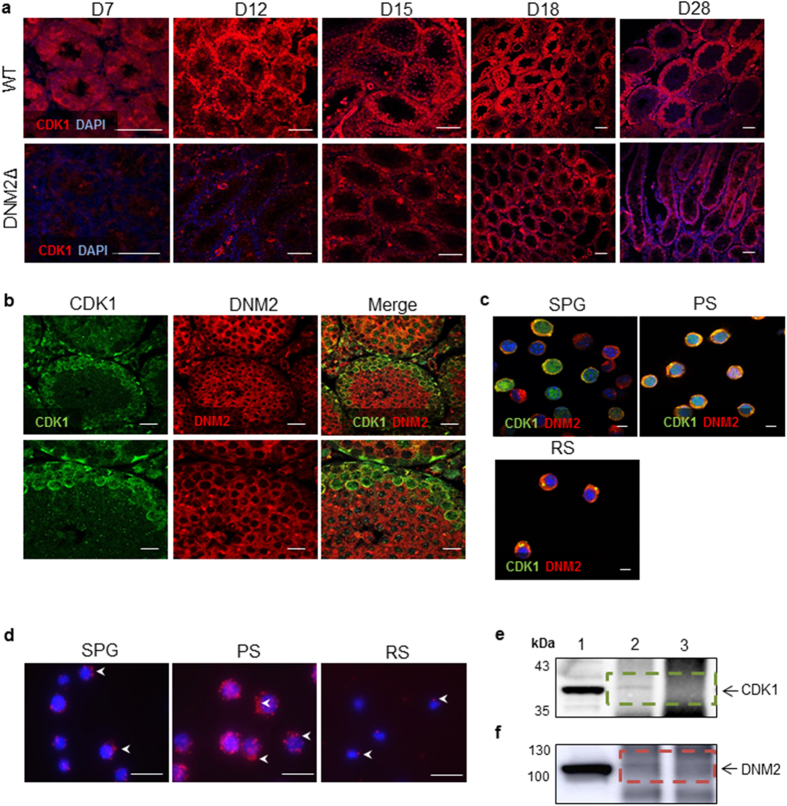
CDK1 as a potential DNM2 phosphorylating kinase in mouse testes. (**a**) Immunofluorescent staining of CDK1 in WT and DNM2Δ testes (Scale bar = 20 μm). (**b**) Dual immunofluorescent staining of fixed adult testis sections probing for CDK1 and DNM2 (Scale bar = 50 μm). (**c**) Isolated SPG, PS and RS were fixed and probed for CDK1 and DNM2. The cells were counterstained with DAPI and visualized using confocal microscopy (Scale bar = 5 μm). (**d**) PLA (Duolink) for CDK1 and DNM2 on isolated SPG, PS and RS. Red foci (arrows) show areas of detection of protein interaction. Cells are counterstained with DAPI (Scale bar = 20 μm). (**e**) Immunoblot for CDK1 immunoprecipitation (IP) using DNM2 as bait (band observed at 34 kDa): lysate (lane 1) contains precleared lysate proteins (positive control), DNM2 IP (lane 2) and control lane (lane 3) represents rabbit IgG IP. (**f**) Immunoblot for reciprocal DNM2 IP (band observed at ~100 kDa): lysate (lane 1) contains precleared lysate proteins (positive control), CDK1 IP (lane 2) and control lane (lane 3) represents eluted proteins for rabbit IgG IP. Dotted boxes highlight that in both IPs target protein was pulled down (absent from control).

Since DNM2 has a crucial role in meiosis, we sought to investigate if DNM2 activity was regulated through cyclic phosphorylation/dephosphorylation events, as previously demonstrated in mature spermatozoa^2^,^29^. Specifically, we focused on CDK1 and calcineurin, the key kinase and phosphatase that mediate dynamin function in somatic cells^8^,^30^. Through immunocytochemistry we confirmed the expression of CDK1 within the SPG and spermatocytes of WT mice from D7. However, in the testes of DNM2Δ mice, the onset of CDK1 expression was delayed until D15 (Fig. 6a). Dual immunofluorescent staining demonstrated strong co-localization of CDK1 and DNM2 in the cytoplasm of SPG and PS in WT testes (Fig. 6b), suggestive of an interaction between CDK1 and DNM2 during mitosis and meiosis I of spermatogenesis. Further support for this putative interaction was afforded by PLA co-localization of the two proteins in the cytoplasm and sub-plasma membrane of isolated SPG and PS (Fig. 6c). A close association was detected between DNM2 and CDK1 (<40 nm) initially in SPG before increasing in PS and again becoming restricted to a single foci in the developing acrosome of RS (Fig. 6d), aligning with the immunocytochemistry results. The use of a co-immunoprecipitation (IP) strategy confirmed this interaction, with a DNM2-specific antibody being employed to successfully pulldown CDK1 from whole testis lysate (Fig. 6e). Similarly, the reciprocal IP with a CDK1-specific antibody was also able to capture and elute the DNM2 protein (Fig. 6f). The specificity of both IP assays was confirmed through the incorporation of an antibody only negative control (Fig. 6f; lane 3) and mouse brain lysate as a positive control (Fig. 6f; lane 1). Importantly, no interaction was observed between DNM2 and other potential phosphorylating kinases including CDK2, CDK5 or GSK3 (Fig. S3).

### Calcineurin (PPP3CA) as a potential DNM2 dephosphorylating phosphatase in mouse testes

**Figure 7:**
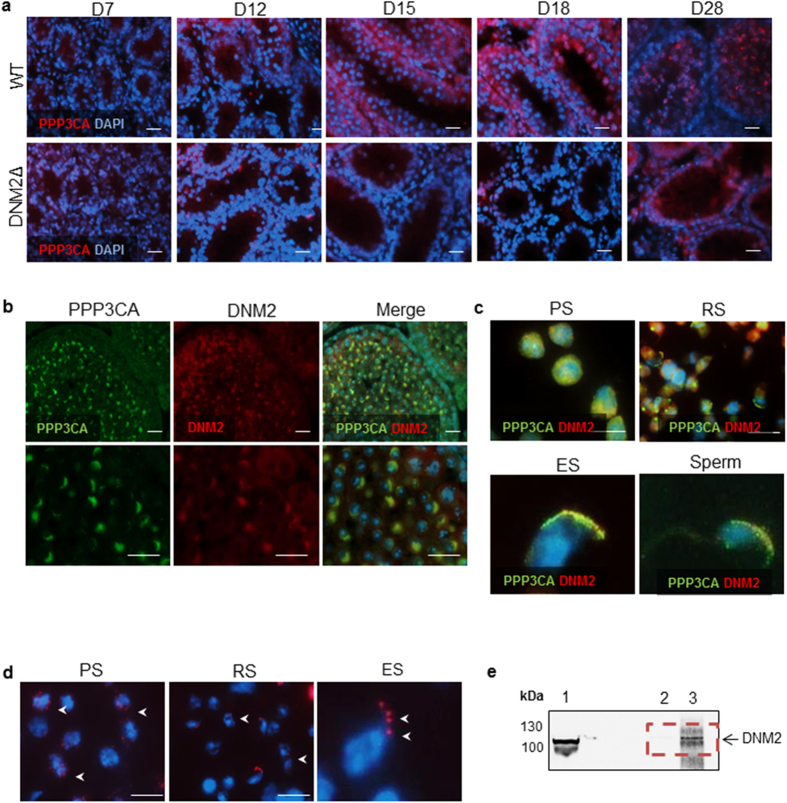
Calcineurin (PPP3CA) as a potential DNM2 targeting phosphatase in mouse testes. (**a**) Immunofluorescent staining of PPP3CA in WT and DNM2Δ testes (Scale bar = 20 μm) (**b**) Dual immunofluorescent staining of fixed adult testis sections probing for PPPC3A and DNM2 (Scale bar = 20 μm). (**c**) Isolated PS, RS, elongating spermatids (ES) and sperm were fixed and probed for PPP3CA and DNM2 protein expression. The cells were counterstained with DAPI and visualized using confocal microscopy (Scale bar = 20 μm). (**d**) PLA (Duolink) for PPP3CA and DNM2 on isolated PS, RS and elongating spermatids (ES). Red foci (arrows) show areas of detection of protein interaction. Cells are counterstained with DAPI (Scale bar = 20 μm). (**e**) Immunoblot for DNM2 immunoprecipitation (IP) using PPP3CA as bait (band observed at ~100 kDa): lysate (lane 1) contains precleared lysate proteins (positive control), control lane (lane 2) represents rabbit IgG IP and DNM2 IP (lane 3). Dotted box highlights that in the IP the target protein was pulled down (absent from control).

Although the role of PPP3CA has been extensively studied in neurons, and its relationship with DNM2 has been previously demonstrated in HeLa cells^8^, no studies have been performed on the expression and role of PPP3CA within the testes. Using immunofluorescence we examined whether PPP3CA is present within the testis, and if it is specifically associated with DNM2 during spermatogenesis. The localization pattern observed in Fig. 7a indicates that PPP3CA is present in D7 mouse testes at low levels in SPG (Fig. 7a). This expression increased at D15 and was strongly associated with RS by D18, and with the developing acrosome at D28. In the testes of DNM2Δ mice however, we failed to see this pattern emerge due to the loss of post-meiotic germ cells within the tubules. Instead, PPP3CA remained ubiquitously expressed in the remaining SPG. In order to determine if PPP3CA is associated with DNM2 during spermatogenesis, we performed dual immunofluorescent labelling. DNM2 and PPP3CA begin to co-localize within the sub plasma membrane region of PS (Fig. 7b,c). As the cells mature into RS, this co-localization becomes restricted to the developing acrosome as the cells elongate (Fig. 7b,c). PLA was performed to determine if there was a close association between DNM2 and PPP3CA (<40 nm), with a positive signal observed around the periphery of isolated PS, and again becoming more closely associated with the developing acrosome in RS (Fig. 7d). In elongating spermatids the association between these two proteins was observed only in the acrosomal cap (Fig. 7d). Finally, an IP strategy was adopted whereby adult testis lysates were precipitated with anti-PPP3CA antibody, and subsequently examined for the presence of DNM2 protein (Fig. 7e). This technique effectively isolated the target protein DNM2 using PPP3CA as bait, thus supporting an interaction between the two proteins.

## Discussion

Since its identification more than two decades ago as a GTPase that co-purified with microtubules in the brain^5^,^31^, dynamin has been established as a key regulator in the formation and fission of endocytic vesicles. The dynamin family has been widely studied in relation to its regulation of neural pathways^14^. However, more recent studies have also demonstrated that dynamin is present within mammalian testes and spermatozoa, where it plays a critical role in successful acrosomal exocytosis during fertilisation^2^,^29^. While these studies demonstrated the presence of DNM1 and DNM2 in the developing acrosome of male germ cells, the importance of these two proteins during spermatogenesis was not investigated.

Previous studies have implicated the three ‘classical’ dynamins in essential roles within the mammalian testis, including maintenance and turnover of the blood-testis-barrier^32^, the phagocytosis of residual bodies^33^,^34^, and regulation of acrosome formation and maturation^2^,^29^. In fulfilling these roles, there appears to be both distinct and redundant functions for the three dynamin members. The latter may in part be attributed to a conserved modular domain structure and an overall identity of 80% among these three DNM isoforms. Nevertheless, each isoform does display distinct spatial and temporal patterns of expression patterns across a number of tissues^3^,^10^,^11^,^13^, and they are also characterized by unique protein-protein interactions^35^,^36^,^37^. Within the testis, DNM1 and DNM2 have been previously shown to localize predominantly to the cytoplasm of PS before becoming restricted to the developing acrosome in round and elongating spermatids. In contrast, DNM3 displays only a modest punctate pattern of expression in these cell types^2^. In this study we observed additional expression of DNM2 within the SPG and of the early postnatal testis. However, the ensuing depletion of the developing germ cells clearly demonstrates that neither DNM1, nor DNM3 are capable of compensating for DNM2 and that these enzymes are unlikely to have redundant roles within the germline.

The impact that loss of DNM2 has on germ cell development is clear through the significant reduction or loss of cell cycle markers in the testes of DNM2Δ mice, suggesting that a large proportion of SPG are failing to correctly complete and exit mitosis during the first wave of spermatogenesis. A similar phenotype has also been documented in immortalized embryonic stem cells bearing a targeted deletion for DNM2^12^. These mutant cells display retarded growth trajectory, but interestingly, show no accompanying defects in cell cycle progression or increases in multi-nucleated cells and apoptotic markers. Further, while both control and DNM2Δ cells progressed through mitosis with similar kinetics, the time to complete midbody separation was significantly longer in DNM2Δ cells^38^. As an additional defect in mitotic exit, the total SPG stem cell population is also reduced by DNM2 ablation, with SPG markers SALL4 and PLZF severely reduced by D12. Furthermore, in those SPG that persist up to D28, only very low levels of SALL4 expression were detected and PLZF appeared to have been lost completely. These findings are of significance as the loss of SALL4 in the testis is known to result in failure to establish a robust stem cell pool^39^ and similarly, mice lacking PLZF undergo a progressive loss of SPG with age^40^.

The phosphorylation of H2AX to γH2AX indicates the formation of DNA DSBs^23^, and is therefore frequently used as a marker of DNA damage. However, since the process of meiotic recombination in mice is initiated by programmed DSBs^24^, the presence of γH2AX staining serves as a robust indicator of normal meiotic function. The significant loss of γH2AX staining in meiotic spermatocytes in DNM2Δ testes indicates that the few SPG that successfully exit mitosis, are subsequently failing to successfully transition into meiosis. This notion is further supported by the concurrent loss of YBX2, which has dual functions as a co-activator of transcription in male germ cells, as well as in the translational repression and storage of paternal mRNAs in spermatocytes and spermatids. Loss of this DNA RNA-binding protein in mutant mice results in post-meiotic arrest during spermiogenesis and infertility^41^. In addition, analysis of the meiotic spreads indicate a loss of both γH2AX and SCP3 in the germ cells of DNM2Δ mice. SCP3 is an essential component of the synaptonemal complex, a structure that functions as a key regulator of meiotic chromosome behaviour^42^,^43^,^44^, with the selective ablation of this protein leading to failed chromosomal synapses in primary spermatocytes and subsequent infertility^45^.

Following failed progression into meiosis, male germ cells from DNM2Δ mice underwent apoptosis, coinciding with the zygotene-pachytene transition at D12 in WT testes. The timing of this germ cell loss correlates with a phenomenon termed the ‘pachytene checkpoint’, which prevents exit from the pachytene stage of meiotic prophase I if meiotic recombination and chromosome synapse are incomplete^43^. Several important meiotic checkpoint factors have been characterised, and the loss of these key proteins during recombination results in apoptosis^45^,^46^,^47^. Indeed, the pachytene checkpoint is thought to be the result of multiple checkpoint mechanisms converging to cause apoptosis, including failed silencing of the sex chromosome, disruption of Sertoli-cell developmental signals or defects during spermatogenesis^48^,^49^.

It is well established that dynamin function is regulated by cyclic phosphorylation and dephosphorylation events. In neural cells, the phosphorylation of DNM1 on serine residues 774 and 778 is driven by cyclin-dependent kinase 5 (CDK5)^50^,^51^ and on Ser774 by GSK3^52^. In contrast, DNM2 is phosphorylated on Ser764 (a site orthologous to DNM1 Ser778) by CDK1 and CDK2 in HeLa cells^8^. Interestingly, dephosphorylation appears to be driven by the Ca^2+^/calmodulin-dependent phosphatase calcineurin (PPP3CA) in both these cell types^8^,^53^. In agreement with these studies, we demonstrate that both CDK1 and PPP3CA co-localize with DNM2 in the mouse testis in a temporally and spatially regulated manner. CDK1 is most highly expressed in SPG and PS, with expression becoming restricted to the developing acrosome in RS. Interestingly DNM2 ablation in the testis resulted in suppressed CDK1 expression until D15 compared to WT controls. In addition to acting as a potential DNM2-targeting kinase, CDK1 has been shown to be essential for driving the cell cycle during mitosis^54^,^55^, directing resumption of meiosis in oocytes^56^, as well as regulating the timing of kinetochore-microtubule attachment during meiosis I^57^.

**Figure 8:**
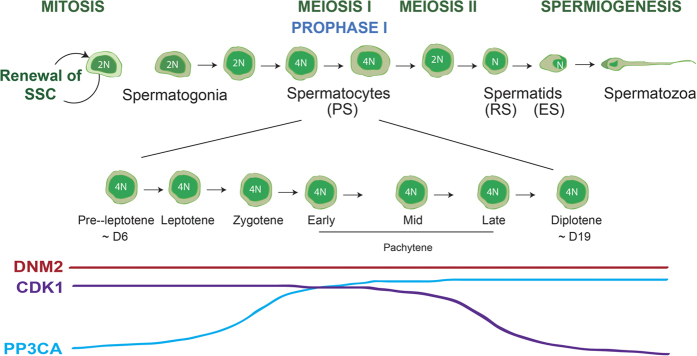
The presence and regulation of DNM2 during mammalian spermatogenesis. Spermatogenesis in the mouse model begins with the mitotic division of spermatogonial stem cells. These proliferating cells then enter meiosis I to become spermatocytes, followed by RS in meiosis II. Spermiogenesis promotes elongation of the RS into morphologically mature sperm. Throughout this process, DNM2 expression remains constant, with the potential regulating kinase, CDK1, expression high in early stage germ cells, and the regulating phosphatase, PPP3CA, high in late stage germ.

Calcineurin is a Ca^2+^/calmodulin-dependent serine phosphatase that has a number of biological functions and has also been previously identified in testis^58^ and spermatozoa^59^. In contrast to a previous study performed in mice which localised PPP3CA exclusively to the nuclei of round and elongating spermatids^58^, we demonstrate expression in the cytoplasm of pachytene spermatocytes, which subsequently becomes restricted to the developing acrosome in round and elongating spermatids, and is highly expressed in the acrosome of mature spermatozoa. This localization would be conducive with a regulatory role in acrosomal biogenesis and exocytosis, and accords with previous studies demonstrating that both dynamin and PPP3CA play key roles in the progression of the acrosome reaction^2^,^29^,^60^. Based on these data we propose that DNM2 function within the testis is regulated in a cyclic manner involving successive waves of phosphorylation/dephosphorylation as has been reported for DNM1 in neural cells^53^,^61^,^62^,^63^. Thus, CDK1 associates with DNM2 in early and mid-stage germ cells and regulates DNM2 activity during mitosis and meiosis, while PPP3CA becomes associated in the late stages of spermatogenesis in order to potentially regulate DNM2 function in the acrosome of mature cells (Fig. 8).

Despite being more thoroughly studied in the context of regulating endo-and exocytosis, the dynamin family of proteins were originally identified as microtubule-binding proteins^5^. Accordingly, DNM2 has since been demonstrated to play significant roles in centrosome cohesion^7^ and regulation of cytokinesis in HeLa cells^8^,^64^. Importantly, phosphorylation of DNM2 by CDK1 has been shown to reduce its ability to bind to microtubules^8^. In studies of mouse models simulating Charcot-Marie-Tooth neurological disease, DNM2 has also been implicated in the maintenance of microtubule stability and microtubule-dependent membrane transport^6^. Moreover, DNM2 is associated with the actin cytoskeleton during female meiosis in mature MII eggs, whereby it regulates spindle migration and cytokinesis^65^. Although additional studies would be required for confirmation, it is thus considered feasible that DNM2 could fulfil an analogous role in the regulation of spindle formation and/or function during the first meiotic phase of spermatogenesis. Such activity could be mediated via direct interaction with the microtubules, or alternative through an indirect association with the actin cytoskeleton.

In conclusion we demonstrate that DNM2 is essential for successful spermatogenesis in the mouse model. We propose that DNM2 holds several key functions during the spermatogenic cycle that centre on its capacity to regulate meiosis. Furthermore, we provide evidence that DNM2 activity is regulated by the spatial and temporal expression of the phosphorylating kinase CDK1, and the phosphatase PPP3CA.

## Methods

Unless otherwise stated, all reagents were obtained from Sigma-Aldrich (St Louis, MO, USA) unless otherwise specified. The dynamin inhibitor Dyngo 6a (a trademark of Children’s Medical Research Institute and Newcastle Innovation Ltd) was synthesized as previously described^66^,^67^ and prepared as a 100 mM stock solution in DMSO prior to serial dilution into appropriate media. All experiments were performed using 3 biological replicates n = 3 times.

### Generation of DNMΔ Mice

All research animals were handled, monitored and euthanized in accordance with the NSW Animal Research Act 1998, NSW Animal Research Regulation 2010 and the Australian Code for the Care and Use of Animals for Scientific Purposes 8^th^ Ed. as approved by the University of Newcastle Animal Care and Ethics Committee. DNM2^loxP/loxP^, DDX4Cre mice were obtained from the Jackson Laboratory (Bar Harbor, ME, USA) and maintained on a C57BL/6 background. DDX4Cre/DNM2^loxP/wt^ males were used as studs with DNM2^loxP/loxP^ females to produce null (DDX4Cre; DNM2^loxP/loxP^), heterozygous (DDX4Cre; DNM2^wt/loxP^) and control littermates (DNM2^loxP/loxP^; DNM2^loxP/wt^). Genotyping primers were generated and used as previously described [DDX4Cre^68^; DNM2^12^].

### Germ Cell Isolation

Pachytene spermatocytes and round spermatids were isolated by loading two adult (>PND60) dissociated testes onto a 2–4% continuous bovine serum albumin (BSA) gradient, as previously described^69^. Following separation, isolated cells were washed in filtered, sterile PBS and used for various applications.

### RNA Extraction

Total RNA isolation was performed using two rounds of a modified acid guanidinium thiocyanate–phenol–chloroform protocol followed by isopropanol precipitation^70^.

### Reverse Transcription PCR (RT-PCR) and Quantitative PCR (qPCR)

Reverse transcription was performed as described^71^. Total RNA was DNase treated prior to reverse transcription to remove genomic DNA. Reverse transcription reactions were verified by *Β-actin* RT-PCR using cDNA amplified with GoTaq Flexi (Promega; Madison, WI, USA). QPCR was performed using SYBR Green GoTaq qPCR master mix (Promega) according to manufacturer’s instructions on LightCycler 96 SW 1.0 (Roche; Castle Hill, NSW, Australia). Primer sequences have been supplied (S1 Table). Reactions were performed on cDNA equivalent to 50 ng of total RNA and carried out for 45 amplification cycles. SYBR Green fluorescence was measured after the extension step at the end of each amplification cycle and quantified using LightCycler Analysis Software (Roche).

### Immunofluorescence (IF)/Immunocytochemistry (ICC)

Whole mouse isolated testes were placed in Bouin’s fixative for 12–24 h, washed in 70% ethanol, paraffin embedded and serially sectioned (4 μm thick) throughout the entire testes, with every 4th slide counterstained with haematoxylin and eosin. Antibodies outlined in Table S2 were used to probe testis tissue sections using the same protocol. Testis sections were deparaffinised, rehydrated, and permeabilized via heat-mediated antigen retrieval. Sections were blocked in 3% BSA/PBS for 1.5 h at room temperature. Sections were incubated with primary antibody of choice for 1 h at room temperature in 1% BSA/PBS. After washing in PBS containing 0.05% Triton X-100, sections were incubated with the appropriate fluorescent conjugated secondary antibodies (IgG Alexa Fluor Conjugate 633/594/488, Invitrogen, 1:200 dilution) for 1 h. Sections were counter-stained with 4′-6-Diamidino-2-phenylindole (DAPI) and mounted in Mowiol. Images were acquired using an Olympus FV1000 confocal microscope (Olympus, Japan) or Axio Imager A1 epifluorescence microscope (Carl Zeiss, USA) with Olympus DP70 camera. Data analysis was performed using ImageJ (National Institutes of Health, MD, USA).

### Proximity Ligation Assay (Duolink PLA)

PLA was performed as previously described^72^ using the Duolink *in situ* kit (OLINK Biosciences, Sweden). Antibodies outlined in Table S2 were used.

### TUNEL Analysis

Bouin’s fixed testis sections were deparaffinised, rehydrated, and permeabilized via heat-mediated antigen retrieval. These were treated with 20 μg/ml Proteinase K for 15 min and TUNEL analysis was performed using an Apoptag Peroxidase *In Situ* Apoptosis Detection Kit (Merck Millipore, USA) according to the manufacturer’s instructions. Slides were counter-stained with DAPI, mounted in Mowiol and observed using an Axio Imager A1 epifluorescence microscope (Carl Zeiss, USA) with Olympus DP70 camera.

### Meiotic Spreads

Pachytene spermatocytes were isolated via density gradient sedimentation as described above. The isolated cell fraction was centrifuged at 500 × g and resuspended in DMEM and 0.5 mM H_2_O_2_. A solution of 1% paraformaldehyde (pH 9.2) and 0.05% Triton X-100 was spread on a glass slide, followed by a droplet of cell suspension. The slide was air dried for 1–2 h, washed for 1 min in 0.4% Photo-Flo 200 solution (Kodak) and air dried for a further 1–2 min. Immunocytochemistry was then performed as described above with antibodies outlined in Table S2.

### Immunoblotting

Protein was extracted from cell lysates using 300 μl RIPA lysis buffer (150 mM sodium chloride, 0.5% sodium deoxycholate, 1.0% Triton X-100, 0.1% SDS, 50 mM Tris, pH 8.0, Protocease protease inhibitor (GBiosciences St. Louis, MO, USA) per 5 mg of cells or tissue. Protein concentration was estimated using a Pierce BCA Protein Assay Kit (Thermo Scientific). Immunodetection was conducted as previously described^69^, using antibodies outlined in Table S2. Anti-mouse/rabbit HRP-conjugated secondary antibodies were used (DAKO, DK) and detection performed using ECL Reagents (GE Healthcare, UK). Membranes were stripped of primary and secondary antibodies to allow re-probing using Western Re-Probe (G-Biosciences), according to the manufacturer’s instructions. Labelled proteins were recorded using a cooled charge-coupled device camera system (Fuji-LAS-4000, Fujifilm Life Science Systems; Tokyo, Japan).

### Immunoprecipitation (IP)

Protein-IP was performed on whole adult ovaries prepared in protein-IP lysis buffer (10 mM CHAPS, 10 mM HEPES, pH 7.0, 137 mM NaCl, 10% Glycerol, 0.01% Protocease protease inhibitor). IP was performed using 10 μg of primary antibody bound to Dynabeads Protein G (Invitrogen; Carlsbad, CA, USA) as described by the manufacturer. Following antibody binding (Table S2), cross-linking was performed using 2 mM of DTSSP (3,3′-dithiobissulfosuccinimidylpropionate). Antibody bound beads were incubated with lysate at 4 °C overnight and bound proteins were eluted using a denaturing protocol. The captured proteins were separated by SDS PAGE and transferred to nitrocellulose membranes in preparation for immunoblotting.

### Statistical Analyses

Statistical analysis was performed using JMP11 analysis software (SAS, Buckinghamshire, UK). All experiments were replicated with a minimum of 3 independent biological samples. The majority of datasets presented a positively or negatively skewed distribution for which non-parametric Wilcoxon/Kruskal-Wallis testing was administered. In figures, * are used to denotes statistical significance, specifically; ***p < 0.001, **p < 0.01 *p < 0.05.

## Additional Information

**How to cite this article**: Redgrove, K. A. *et al*. Dynamin 2 is essential for mammalian spermatogenesis. *Sci. Rep.* **6**, 35084; doi: 10.1038/srep35084 (2016).

## Supplementary Material

Supplementary Information
