# Supplementary material for: Dynamin 2 is essential for mammalian spermatogenesis
Source: Sci Rep. 2016 Oct 11;6:35084. doi: 10.1038/srep35084 (PMC5057128; doi:10.1038/srep35084)
Supplement: Supplementary Information [file srep35084-s1.pdf]

Dynamin 2 is essential for mammalian spermatogenesis

Kate A. Redgrove[1,3*], Ilana R. Bernstein[1,3], Victoria J. Pye[1,3], Bettina P. Mihalas[1], Jessie M. Sutherland[2], Brett Nixon[1,3], Adam McCluskey[1,3], Phillip J. Robinson[4], Janet E. Holt[1,3], Eileen A. McLaughlin[1,3,5].

Supplementary Information

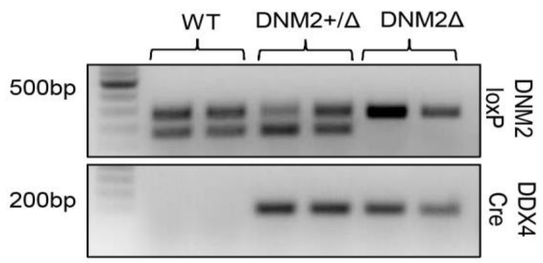

Fig S1: Genotyping of WT, DNM2+/Δ and DNM2Δ animals. Upper panel shows the detection of the floxed DNM2 allele (DNM2Δ) as evidenced by the single amplicon at 258bp. Heterozygote (DNM2+/Δ) has two amplicons at 258 and 192bp. Wildtype (WT) has a single amplicon at 192bp. The lower panel shows the expression of the DDX4 Cre Recombinase as evidenced by the single band at 100bp. WT is represented by the absence of a band.

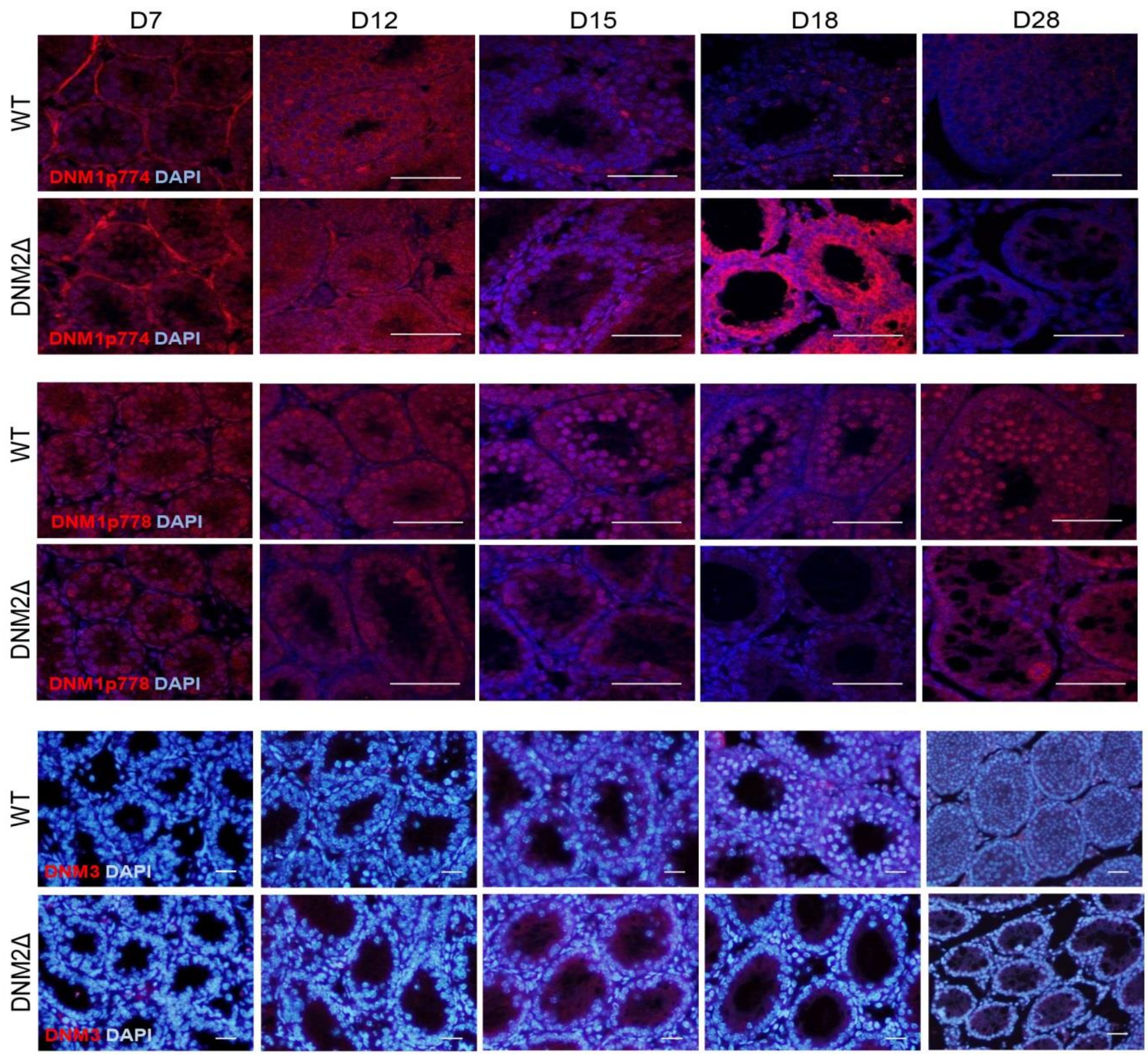

Fig. S2: Expression of DNM1p774, DNM1p778 and DNM3 in WT and DNM2Δ testes. Fixed tissue sections from D7, D12, D15, D18 and D28 WT and DNM2Δ mice testes were probed for (a) DNM1p774, (b) DNM1p778 or (c) DNM3 protein expression using immunofluorescence. The sections were counterstained with the nuclear marker DAPI and visualised using confocal microscopy (a & b) (Scale bar=50μm) or epifluorescence microscopy (c) (Scale bar=20μm).

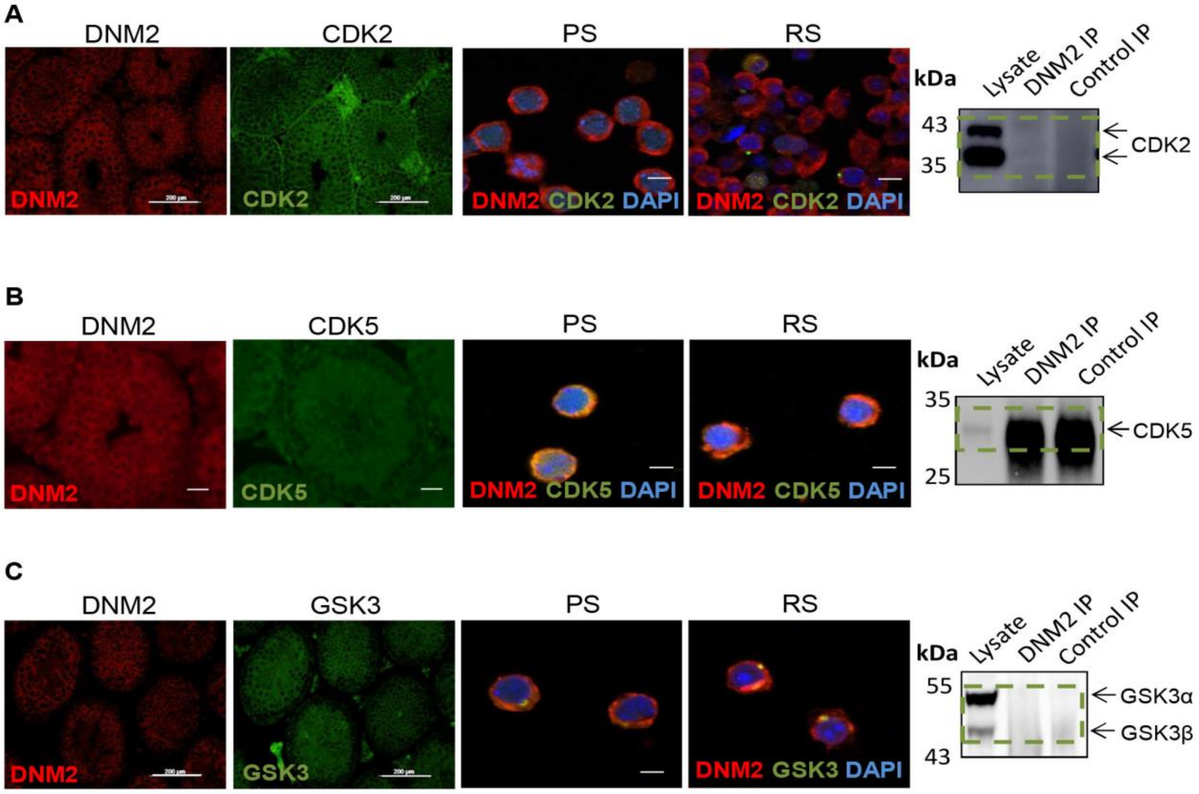

Fig. S3: The expression of DNM2 and potential interacting kinases in mouse testes. (a) Dual immunofluorescence probing for DNM2 and CDK2 in WT adult testes, and isolated PS and RS. Sections and cells were counterstained with the nuclear marker DAPI and visualized using confocal microscopy. Immunoblot for CDK2 immunoprecipitation (IP) using DNM2 as bait (no bands observed at ~34 and 38 kDa): lysate (lane 1) contains precleared lysate proteins (positive control), DNM2 IP (lane 2) and control lane (lane 3) represents rabbit IgG IP (Scale bar IF=200µm; ICC=5µm). (b) Separate immunofluorescence probing for DNM2 and CDK5 in WT adult testes, and isolated PS and RS. All sections and cells were counterstained with the nuclear marker DAPI and visualized using confocal microscopy. Immunoblot for CDK5 immunoprecipitation (IP) using DNM2 as bait (no clear band observed at ~31kDa): lysate (lane 1) contains precleared lysate proteins (positive control), DNM2 IP (lane 2) and control lane (lane 3) represents rabbit IgG IP (Scale bar IF=20µm; ICC=5µm). (c) Dual immunofluorescence probing for DNM2 and GSK3 in WT adult testes,

and isolated PS and RS. Immunoblot for GSK3 immunoprecipitation (IP) using DNM2 as bait (no clear bands observed at ~51 (α) or 47 (β) kDa): lysate (lane 1) contains precleared lysate proteins (positive control), DNM2 IP (lane 2) and control lane (lane 3) represents rabbit IgG IP DNM2 & GSK3 (Scale bar IF=200μm; ICC=5μm).

Table S1. QPCR and genotyping primers.

Primer	Forward Sequence 5'→ 3'	Reverse Sequence 3'→ 5'
Cyclophilin A qPCR	CGTCTCCTTCGAGCTGTTT	ACCCTGGCACATGAATCCT
DNM2 qPCR	ACATTGAGCAGTCGTACATC	TCTCCCCCTGATTGGGTAT
DNM2flox genotyping	CCCTGCTAGTGACCTTTCTTGA	GCAGGAAGACACACAACTGAAC
DDX4Cre genotyping	GTGAAACAGCATTGCTGTCAC TT	GCGGTCTGGCAGTAA AAACTAT

Table S2. Primary antibodies list.

Antibody Name	Manufacture	Catalogue Number	Dilution Used
DNM1p774	Sigma	SAB4503866	1:100
DNM1p778	Abcam	ab18101	1:100
DNM2	Thermo Scientific	PA5-19800	1:200
DNM2	Santa Cruz	sc-6400	1:100
CDK1	Millipore	ABE1403	1:200
YBX2	Abnova	PAB19295	1:200
MKI67	Abcam	ab15580	1:200
PLZF	Gift from Dr. Robin Hobbs		1:100
SALL4	Abcam	ab29112	1:200
CCND1	Abcam	ab16663	1:200
ERCC1	Bioworld	BS1111	1:100
γH2AX	Abcam	ab26350	1:200
PPP3CA (CaN)	Abcam	ab3673	1:200
WT1	Santa Cruz	sc192	1:100
SCP3	Novus Biologicals	NB300-231	1:200
GSK3	Millipore	05-412	1:100
CDK2	Abcam	ab6538	1:100
CDK5	Santa Cruz	sc-173	1:100
